# Microvesicles derived from human Wharton’s Jelly mesenchymal stromal cells ameliorate renal ischemia-reperfusion injury in rats by suppressing CX3CL1

**DOI:** 10.1186/scrt428

**Published:** 2014-03-19

**Authors:** Xiangyu Zou, Guangyuan Zhang, Zhongliang Cheng, Deming Yin, Tao Du, Guanqun Ju, Shuai Miao, Guohua Liu, Mujun Lu, Yingjian Zhu

**Affiliations:** 1 Department of Urology, Shanghai First People’s Hospital, School of Medicine, Shanghai Jiao Tong University, Shanghai, P.R. China; 2 Department of Urology, Ningbo No.2 Hospital, Ningbo, P.R. China; 3 Shanghai Key Laboratory of Tissue Engineering, Tissue Engineering Center, School of Medicine, Shanghai Jiao Tong University, Shanghai, P.R. China; 4 Department of Urology, Henan Provincial People’s Hospital, Zhengzhou, PR China; 5 Department of Urology, Ninth People’s Hospital, School of Medicine, Shanghai Jiao Tong University, Shanghai, P.R. China

## Abstract

**Introduction:**

Studies have demonstrated that mesenchymal stromal cells (MSCs) could reverse acute and chronic kidney injury by a paracrine or endocrine mechanism, and microvesicles (MVs) have been regarded as a crucial means of intercellular communication. In the current study, we focused on the therapeutic effects of human Wharton-Jelly MSCs derived microvesicles (hWJMSC-MVs) in renal ischemia/reperfusion injury and its potential mechanisms.

**Methods:**

MVs isolated from conditioned medium were injected intravenously in rats immediately after ischemia of the left kidney for 60 minutes. The animals were sacrificed at 24 hours, 48 hours and 2 weeks after reperfusion. The infiltration of inflammatory cells was identified by the immunostaining of CD68+ cells. ELISA was employed to determine the inflammatory factors in the kidney and serum von Willebrand Factor (VWF). Tubular cell proliferation and apoptosis were identified by immunostaining. Renal fibrosis was assessed by Masson’s tri-chrome straining and alpha-smooth muscle actin (α-SMA) staining. The CX3CL1 expression in the kidney was measured by immunostaining and Western blot, respectively. *In vitro*, human umbilical vein endothelial cells were treated with or without MVs for 24 or 48 hours under hypoxia injury to test the CX3CL1 by immunostaining and Western blot.

**Results:**

After administration of hWJMSC-MVs in acute kidney injury (AKI) rats, renal cell apoptosis was mitigated and proliferation was enhanced, inflammation was also alleviated in the first 48 hours. MVs also could suppress the expression of CX3CL1 and decrease the number of CD68+ macrophages in the kidney. In the late period, improvement of renal function and abrogation of renal fibrosis were observed. *In vitro*, MVs could down-regulate the expression of CX3CL1 in human umbilical vein endothelial cells under hypoxia injury at 24 or 48 hours.

**Conclusions:**

A single administration of MVs immediately after ischemic AKI could ameliorate renal injury in both the acute and chronic stage, and the anti-inflammatory property of MVs through suppression of CX3CL1 may be a potential mechanism. This establishes a substantial foundation for future research and treatment.

## Introduction

Ischemia/reperfusion (I/R) injury is a major cause of intrinsic acute kidney injury (AKI), which has emerged as the most common condition in hospitalized patients and continues to result in a high mortality rate [1]. Ischemia/reperfusion injury (IRI)-induced AKI may result in proliferation of fibroblasts and excessive deposition of extracellular matrix [2] and has been recognized as a major contributor to end-stage kidney disease [3]. Therefore, a potent therapeutic intervention for ischemic AKI is imperative.

Inflammation is now believed to play a major role in the pathophysiology of AKI [1]. Various specific inflammatory cells and pro-inflammatory cytokines/chemokines are increased in the kidney in the early phase of injury after an ischemic AKI [4,5]. Among these inflammatory cells, a macrophage is an important mediator in the initiation and extension of ischemic/reperfusion injury [6,7]. The function of macrophages and inflammatory cytokines in tissue repair at different stages is still unclear. However, a previous publication showed that strategies which limit the initial macrophage infiltration or activation can be beneficial for ischemic AKI [8]. SJ Yu *et al*. also found that macrophage depletion using clodronate could protect against ischemic AKI [9]. Further studies indicated that knockout or neutralizing against adhesion molecule-1 in mice also prevents macrophage recruitment and the expression of inflammatory cytokines, which consequently induces less severe damage in renal disease models [10,11]. The transmigration of macrophages across the vascular endothelium into tissues depends upon the influence of various factors, including adhesion molecules, chemokines and their receptors [12]. CX3CL1, also called fractalkine, is found mainly expressed on endothelial cells and is a potent chemo-attractant factor for macrophages [13,14]. It has been reported that the up-regulation of CX3CL1 was observed in I/R induced AKI, and CX3CL1/CX3CR1 could promote kidney interstitial fibrosis after IRI [15]. Meanwhile, inhibition of CX3CR1 reduces the macrophages in the injured kidney and has the effects of therapy in AKI [14,16]. These studies indicate that CX3CL1 is an important target molecule in anti-inflammation therapy for ischemic AKI.

Although the kidney has been thought to have a certain capacity for self-repair or regeneration after ischemic AKI, several strategies have been proposed to promote recovery from AKI. Among them, MSCs are thought of as a promising and effective strategy to contribute to kidney repair, although the mechanisms are not very clear now. MSCs display an immune-modulatory activity which is important in the response to tissue injury [17], and the inflammatory suppression role of MSCs in renal IRI was also reported [18]. On the other hand, the paracrine or endocrine mechanisms of MSCs in tissue injury repair have also been proposed recently [19,20]. Microvesicles (MVs) derived from MSCs are recently exploited in regenerative medicine to repair damaged tissues. These membranous structures delivering bioactive molecular contents, such as proteins, mRNAs and micro-RNAs sequences, are described as a novel pathway in cell-to-cell interaction. It has also been reported that MVs derived from MSCs could protect AKI in I/R animal models [21,22]. However, the real mechanisms are unclear and whether the Wharton’s Jelly mesenchymal stromal cells derived microvesicles (hWJMSC-MVs) could play an immunomodulation role in ischemic AKI is unknown.

The objectives of the present study were to investigate the possible therapeutic potential of hWJMSC-MVs to rescue rats from AKI and to clarify the possible mechanism(s) by which hWJMSC-MVs may improve renal function in this ischemic AKI model.

## Materials and methods

### Cell culture

Fresh human umbilical cords that are usually discarded after delivery were obtained with the written consent of the parents. This experiment was approved by the Research Ethics Committee at Shanghai First People’s Hospital-affiliated Shanghai Jiao Tong University (Permit number: 2013KY018). Human umbilical cords were delivered and stored in cold Hank^’^s balanced salt solution (Sigma-Aldrich, St Louis, MO, USA) and then cellular isolation started within 4 h. The hWJMSCs were isolated and identified as described previously [23]. Briefly, with the elimination of umbilical cord vessels, mesenchymal tissues were cut into 1 mm^3^ pieces and then stuck to the substrate of culture plates individually, followed by the addition of low-glucose Dulbecco^,^ Modified Eagle^’^s Medium (DMEM, Gibco BRL Co., USA) containing 10% fetal bovine serum (FBS, Gibco BRL Co., USA) at 37°C in a humidified atmosphere with 5% CO_2_. The medium was changed every two days. After about two weeks^’^ culture, the adherent cells were harvested with 0.25% trypsin (Gibco BRL Co., USA) treatment and sub-cultured. Only cells from three to six passages were used for further experiments.

Human umbilical vein endothelial cells (HUVEC) were isolated from umbilical cord veins by collagenase digestion. In brief, the veins were washed twice with phosphate-buffered saline (PBS), and the HUVECs were flushed out after digestion with 0.1% collagenase (Gibco BRL Co., USA) for 30 minutes at room temperature. HUVECs were seeded onto dishes in EGM-2 medium at 37°C in a humidified atmosphere with 5% CO^2^. The medium was changed every two days. After one week’s culture, the adherent cells were harvested with 0.05% trypsin (Gibco BRL Co., USA) treatment and sub-cultured. Only cells from passages 2 to 4 were used for experiments. Phenotypic analysis of cultured cells was determined by immunofluorescence using CD31 (Abcam, Cambridge, UK) and VE- Cadherin (Abcam).

### Isolation of MVs

MVs were obtained from supernatants of hWJMSCs as was previously described [24]. Briefly, hWJMSCs were cultured in DMEM without FBS and with added 0.5% bovine serum album (BSA) (Sigma-Aldrich) overnight. The viability of the cell cultured overnight was >99% as detected by trypan blue exclusion and no apoptotic cells were detected by terminal transferase-mediated dUTP nick-end labeling (TUNEL) assay. The conditioned medium was collected and stored at -80°C. The medium was centrifuged at 2,000 g for 20 minutes to remove debris, and then ultracentrifuged at 100,000 g in a SW41 swing rotor (Beckman Coulter, Fullerton, CA, USA) for one hour at 4°C. MVs were washed once with serum-free M199 (Sigma-Aldrich) containing 25 mM HEPES (pH = 7.4) and submitted to a second ultracentrifugation in the same conditions. MVs were stored at -80°C for the experiments. To quantify the protein content, the Bradford protein assay kit (P0006, Beyotime Institute of Biotechnology, China) was used in the Bradford assay. We quantified it indirectly according to 5 × 10^5^ MSCs releasing approximately 100 μg MVs overnight. 

### Transmission electron microscopy

MVs were fixed with 2.5% glutaraldehyde in PBS for 2 h, after being washed; MVs were ultra-centrifuged and suspended in 100 uL PBS. A total of 20 uL of MVs was loaded onto a formvar/carbon-coated grid, negatively stained with 3% aqueous phosphor-tungstic acid for one minute and observed by transmission electron microscopy (HITACHI, H-7650, Japan).

### MVs surface marker analysis

Flow cytometry was used to characterize the isolated MVs. MVs were incubated for 30 minutes at room temperature, 5 ml of latex beads were added and incubated for another 30 minutes at 4°C, then washed in 0.5% BSA in PBS and incubated with different antibodies (CD9, CD34, CD44, CD45, CD63, CD73 or with appropriate isotype control IgG. After washing, MV-coated beads were immediately analyzed using a FACS Calibur flow cytometer (Becton Dickinson, FACS Calibur).

### Animal models of unilateral renal IRI

All work involving animals was done in accordance with the animal use protocol enacted by the Institutional Animal Care and Use Committees of School of Medicine, Shanghai Jiao Tong University. All animals used were male SD rats (180 to 200 g), which were housed at a constant temperature and humidity, with a 12:12-h light-dark cycle. Animal models were performed in male SD rats (180 to 200 g) by left ischemia for 60 minutes, Sham-treated animals were treated in a similar manner, except that the renal vessels were not clamped. A total of 100 ug MVs in 1 mL of vehicle (M199, Gibco BRL Co., USA) or a total of 1 ml of vehicle only was administered via caudal vein immediately after reperfusion. The animals were separated into different groups according to different therapeutic procedures. We used at least six rats for each group at different time points: 1) sham-treated rats (n = 18); 2) vehicle-injected IRI rats (n = 18); MVs-injected IRI rats (n = 18). The rats were sacrificed at 24 h, 48 h and 2 weeks, respectively. Blood and kidney samples were collected and submitted for corresponding examination. To trace MVs in the kidneys, the PKH-26 dye (Sigma) kit was used to label the MVs. Then, 100 ug of PKH-26 labeled MVs were injected intravenously into AKI rats, and the unlabeled MVs were used as a control. Rats were sacrificed after 3 h and kidneys were acquired for frozen sectioning. The Cytokeratin 19 antibody (Abcam,) was used for staining the cytoplasm of tubular epithelial cells; Hoechst 33258 dye (Sigma) was added for nuclear staining.

### Renal function

After the right uninjured kidneys of the rats were removed on Day 12, blood samples were obtained for measurement of plasma BUN and creatinine on Day 14. Serum creatinine and BUN were measured by a Beckman Analyzer II (Beckman Instruments, Inc.).

### ELISA analyses of vWF levels in serum and cytokine expression in injured kidneys

For analysis of the level of vWF in serum, samples were frozen at -80°C until analysis and we used a Rat vWF Elisa kit following the manufacturer’s instructions (Eiaab, Hubei, China), and for analysis of TNF-α and IL-10 in injured kidneys, samples were frozen at -80°C until analysis. We used a Rat TNF-α Elisa kit (R & D Systems Inc., USA) and an IL-10 Elisa kit (R & D Systems, Inc., USA) following the manufacturer’s instructions.

### Immunohistochemistry

One portion of the renal tissue was fixed in 4% paraformaldehyde and embedded in paraffin. Sections 4 um-thick were labeled with mouse antibody to rat CD68 (dilution 1:100; Abcam), mouse antibody to rat α-SMA (dilution 1:500; Abcam), rat antibody to human CX3CL1 (dilution 1:250; Abcam), rat antibody to human Ki67 (dilution 1:250; Abcam) followed by horseradish peroxide (HRP)-conjugated secondary antibody using diaminobenzidine (DAB) reagents as substrates and then counterstained with hematoxylin. The numbers of CD68+ cells in the corticomedullary junctions and outer medullas of kidneys were randomly counted in an average of 30 high power fields (HPF). Under 400× magnification, scoring for Ki67-positive cells was carried out by counting the number of positive tubular cell nuclei in 30 random fields from randomly chosen kidney sections for each animal (n = 6 rats, each group). Tubular cell apoptosis was assessed by a TUNEL assay using an *In Situ* Cell Death Detection Kit (Roche, Mannheim, Germany). Kidney sections were screened for tubular cells of positive nuclei under 400× magnification. The apoptotic score was achieved by counting the number of positive nuclei in 30 random fields.

### Hematoxylin and Eosin (H&E) staining

To detect the injury to the kidneys, they were fixed in 4% paraformaldehyde (pH 7.4), gradually dehydrated, embedded in paraffin, cut into 4-μM sections and stained with H&E stain. Histopathology scoring was applied based on a previous study in a blinded fashion [23]. The score was given based on the grading of tubular necrosis, loss of brush border, cast formation and tubular dilatation in 10 randomly chosen, non-overlapping fields (200×) as follows: 0 (none), 1 (≤10%), 2 (11 to 25%), 3 (26 to 45%), 4 (46 to 75%), and 5 (≥76%).

### Masson’s trichromatic staining

The degree of interstitial fibrosis was scored semi-quantitatively on a 0 to 3 scale (0, no lesion; 1, <33% of parenchyma affected by the lesion; 2, 33% to 67% of parenchyma affected by the lesion; 3, >67% of parenchyma affected by the lesion). The scores were assessed by a blinded observer in 100 HPFs (magnification 400×) of parenchyma for each rat (n = 6 rats, each group). The total score was obtained by the addition of all scores, with a maximum score of 300.

### Western blot

Protein concentration was measured with BCA Protein Assay, 30 ug of total protein were electrophoresed on an 8% to 10% SDS-PAGE gel and then transferred onto nitrocellulose membranes (Millipore, USA). Membranes were blocked in 5% non-fat milk in TBS containing 0.1% Tween 20 for 1 h at room temperature, and then each membrane was incubated with a rabbit antibody to rat CX3CL1 (dilution 1:1,000; Abcam), mouse antibody to α-SMA (dilution 1:1,000; Abcam) or a GAPDH antibody overnight at 4°C. After being washed in PBS, each membrane was incubated for one to two hours with a secondary antibody conjugated by peroxidase at room temperature, detected by ECL reagent (Millipore, USA). The density of each band was analyzed by Image-Pro Plus 6.0 software.

### Immunochemistry staining for CX3CL1 expression in HUVECs

HUVECs injured by hypoxia were incubated on chamber slides and exposed to MVs or control medium for 48 hours. Subsequently, the slides were fixed in 4% paraformaldehyde and permeabilized with HEPES-250 Triton × 100 buffer (Sigma). Rat antibody to human CX3CL1 (dilution 1:1,000; Abcam) was used as the primary antibody. Harris hematoxylin was added for nuclear counterstaining.

### RNA extraction and micro-RNA profiling by real-time quantitative PCR

Total RNA was isolated from hWJMSC-MVs by Trizol (Invitrogen, Carlsbad, CA, USA) extraction method. To increase the yield of small RNAs, the RNA was precipitated overnight. RNA concentration and RNA integrity were determined by capillary electrophoresis on an Agilent 2100 Bioanalyzer (Agilent Technologies, Palo Alto, CA, USA). At the time of the study, commercially available primers (designed and synthetized by BioTNT, Shanghai, China) were available for six mature human micro-RNAs (miR-15a, miR-15b, miR-16, miR-195, miR-424 and miR-497). We used these looped primers to profile six mature micro-RNAs by real-time PCR using an Applied Biosystems’ 7900HT real-time PCR instrument. RNA was converted to cDNA by priming with a mixture of looped primers (designed and synthetized by BioTNT, Shanghai, China) using previously published reverse transcription conditions [25]. Each RT reaction contained 1 ug of total RNA, and real-time PCR was performed using standard conditions.

### Statistical analysis

Results from at least three independent experiments are reported as the means ± standard deviation (SD). Statistical analyses were performed using SPSS v19.0. A value of *P* <0.05 was considered to be statistically significant.

## Results

### Isolation and expansion of hWJMSCs

hWJMSCs were isolated and identified as our group previously showed [23]. Briefly, the cells had a spindle-shaped morphology and adhered to plastic surfaces. With FACS analysis, we found that the expression of MSC markers (CD44, CD73, CD90 and CD105) was positive, whereas the expression of hematopoietic markers (CD45, CD34 and CD14) and an endothelial marker (CD31) was negative [23]. In addition, these cells harbored the potential to differentiate toward chondrocytes and osteoblasts [23].

### Isolation and characterization of HUVECs

HUVECs were isolated from umbilical cord vein by collagenase digestion successfully. Immunofluorescence analysis revealed the positive expression of CD31 and VE-Cadherin.

### Extraction and characterization of hWJMSC-MVs

**Figure 1:**
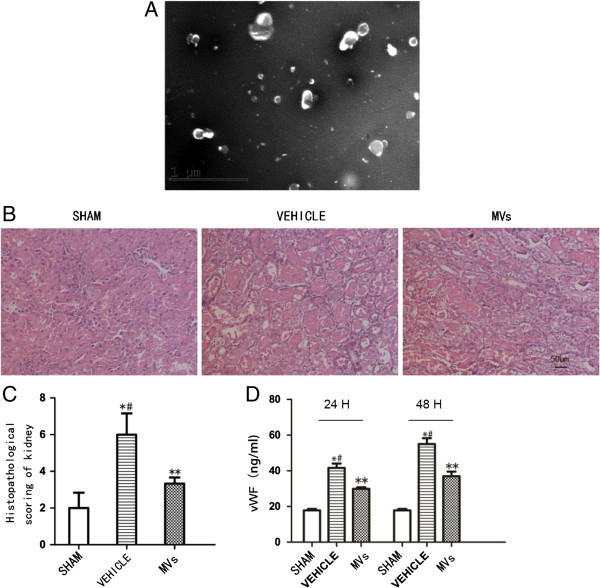
**hWJMSC-MVs observed by transmission electron microscopy and effects in IRI induced AKI in rats. (A)** hWJMSC-MVs were heterogeneous lipid bi-layer vesicles that range from 30 to 500 nm in diameter, and were characterized by cup-shaped morphology (Scale bar = 1 um). **(B)** Representative micrographs of renal histology at 48 h after reperfusion, in sham-operated rats and in MVs rats or vehicle (Original magnification 200×. Scale bar = 50 um). **(C)** Histopathological scoring shows a significant difference between the MV-treated group and vehicle, sham versus vehicle group, MVs versus sham group (n = 6. *P <0.05, sham versus vehicle, *#P* <0.05, MVs versus vehicle, **P <0.05, sham versus MVs). **(D)** vWF in plasma was increased at 24 h and 48 h, respectively, after IRI. However, in the MVs group, there is a significantly decreased vWF level in 24 h and 48 h compared with the vehicle group. All quantitative data are shown as mean ± SD of six serum samples for each experimental condition. (n = 6. *P <0.05, sham versus vehicle; *#P* <0.05, MVs versus vehicle, **P <0.05, sham versus MVs). AKI, acute kidney injury; hWJMSC, human Wharton-Jelly mesenchymal stem cells; IRI, ischemia/reperfusion injury; MVs, microvesicles.

hWJMSC-MVs were successfully isolated and characterized as we previously described [24]. They were heterogeneous lipid bilayer vesicles of approximately 30 to 500 nm in diameter, and characterized as cup-shaped or irregular-shaped (Figure 1A). FACS analysis showed hWJMSC-MVs were positive for some surface-expressed molecules typically expressed by hWJMSCs, such as CD9, CD44, CD63 and CD73 and negative for CD34 and CD45 [24].

### The alleviation of I/R-induced kidney injury by hWJMSC-MVs in early stages

To evaluate the tubular injury level, H&E staining was performed. Lesions were observed in rats at 48 h by H&E staining of kidney tissue slices. The slices showed that numerous necrotic areas of the proximal epithelium had appeared and abundant tubular protein casts had formed in the vehicle and MVs therapy groups. However, the tubular lesions obviously decreased in the MV group compared to the vehicle (Figure 1B). The representation of histological score suggested the same results (Figure 1C). Circulating vWF was measured as a marker of endothelial injury. The serum level of vWF was increased at 24 h and 48 h, compared with sham, after IRI (respectively, 42.13 ± 2.34 ng/ml versus 18.19 ± 1.06 ng/ml, *P* <0.05, and 56.25 ± 3.42 ng/ml versus 18.19 ± 1.06 ng/ml, *P* <0.05). Surprisingly, in the MV group there was a significant reduction of the vWF level at 24 h and 48 h compared with the vehicle group (respectively, 31.18 ± 0.95 ng/ml versus 42.13 ± 2.34 ng/ml *P* <0.05, and 37.70 ± 2.61 ng/ml versus 56.25 ± 3.42 ng/ml, *P* <0.05). Compared with the sham group, the MV group still had a high level (respectively, 31.18 ± 0.95 ng/ml versus 18.19 ± 1.06 ng/ml, *P* <0.05, and 37.70 ± 2.61 ng/ml versus 18.19 ± 1.06 ng/ml, *P* <0.05), which is shown in Figure 1D. Three hours after PKH-26-labeled MVs (red fluorescence) were injected intravenously, red fluorescence was observed in the kidneys of the AKI rats, while there was no red fluorescence detected in the control group (Additional file 1: Figure S1).

### hWJMSC-MVs mitigated the apoptosis and enhanced the proliferation of renal cells

**Figure 2:**
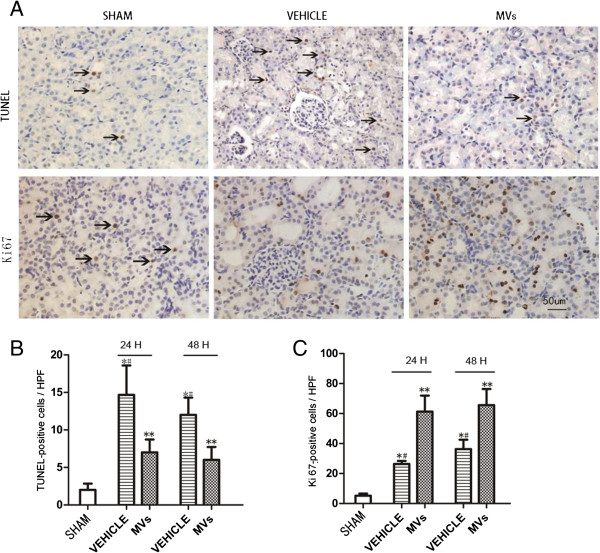
**Effects of hWJMSC-MV administration on the proliferation and apoptosis of tubular epithelium cells. (A)** Representative images of Ki67 and TUNEL immunostaining in the injured kidneys at 48 h after reperfusion treated with hWJMSC-MVs (magnification 200×, Scale bar = 50 um). **(B)** Quantification of TUNEL-positive tubular epithelium cells in the kidney sections at different time points after reperfusion with hWJMSC-MVs. (n = 6,*P <0.05, sham versus vehicle; *#P* <0.05, MVs versus vehicle, **P <0.05, sham versus MVs). **(C)** Quantification of Ki67-positive tubular epithelium cells in the kidney sections at different time points after reperfusion with hWJMSC-MVs. (n = 6, *P <0.05, sham versus vehicle; *#P* <0.05, MVs versus vehicle, **P <0.05, sham versus MVs).

As shown in Figure 2, the number of TUNEL-positive cells in the vehicle and MV groups significantly increased at 24 h and 48 h after IRI compared to the sham group (respectively, 14.8 ± 5.0 versus 2.2 ± 0.8, *P* <0.05 and 12.8 ± 3.0 versus 2.2 ± 0.8, *P* <0.05; 6.9 ± 1.7 versus 2.2 ± 0.8, *P* <0.05 and 6.1 ± 1.5 versus 2.2 ± 0.8, *P* <0.05), while the kidneys of hWJMSC-MV-treated rats exhibited fewer TUNEL-positive cells than the vehicle group (24 h and 48 h, respectively, 6.9 ± 1.7 versus 14.8 ± 5.0, *P* <0.05; 6.1 ± 1.5 versus 12.8 ± 3.0 *P* <0.05). The number of Ki67-positive cells in the vehicle and MV groups significantly increased at 24 h and 48 h after IRI was compared to the sham group (respectively, 26.6 ± 4.7 versus 5.4 ± 2.3 *P* <0.05; 37.9 ± 11.2 versus 5.4 ± 2.3, *P* <0.05; 63.8 ± 13.0 versus 5.4 ± 2.3 *P* <0.05; 65.3 ± 15.0 versus 5.4 ± 2.3, *P* <0.05). Meanwhile, after being treated with hWJMSC-MVs, the number of proliferating cells shown at 24 h and 48 h increased compared with vehicle treatment (respectively, 26.6 ± 4.7 versus 63.8 ± 13.0, *P* <0.05; 37.9 ± 11.2 versus 65.3 ± 15.0, *P* <0.05).

### IRI-induced fibrosis is abrogated by MVs therapy, as well as improvement of renal function in late stage

**Figure 3:**
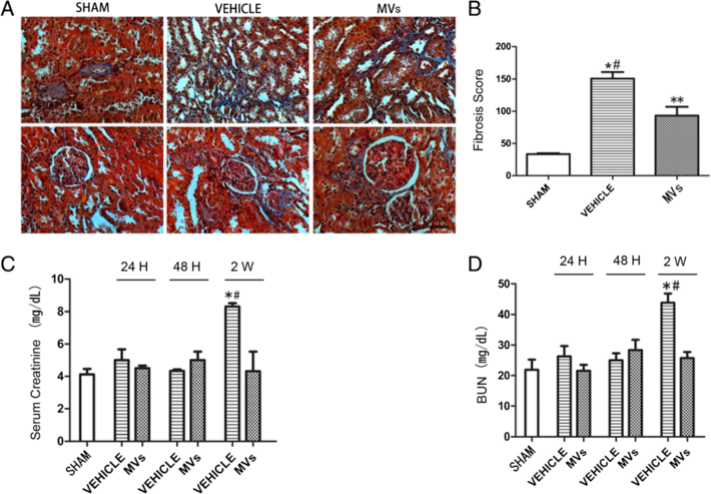
**hWJMSC-MVs alleviate the renal fibrosis in injured kidney tissues and improve renal functions after two weeks. (A)** Representative micrographs of Masson’s tri-chrome staining in tubular interstitial areas. Ischemic injury caused a progressive increase in positive-staining collagen deposition, whereas the MVs group had a remarkable reduction in collagen deposition. (The original magnification is 400×, Scale bar = 50 um). **(B)** Fibrosis score. The highest fibrosis score was achieved by the vehicle group, whereas the MV group had a significantly lower score. The score was obtained by the addition of all scores for collagen staining from 30 random high power fields with a maximum score of 300. All quantitative data are shown as mean ± SD of six kidney samples for each experimental condition. (n = 6, *P <0.05, sham versus vehicle; *#P* <0.05, MVs versus vehicle, **P <0.05, sham versus MVs). **(C, D)** Serum creatinine and BUN levels were measured at the indicated times after injury in the sham, vehicle and MVs groups. The right uninjured kidneys of rats were removed on Day 12, and serum samples collected at different times. Values are the mean ± SD of data with six animals in each group (n = 6, *P <0.05, sham versus vehicle; *#P* <0.05, MVs versus vehicle). hWJMSC-MVs, human Wharton-Jelly MSCs derived microvesicles.

**Figure 4:**
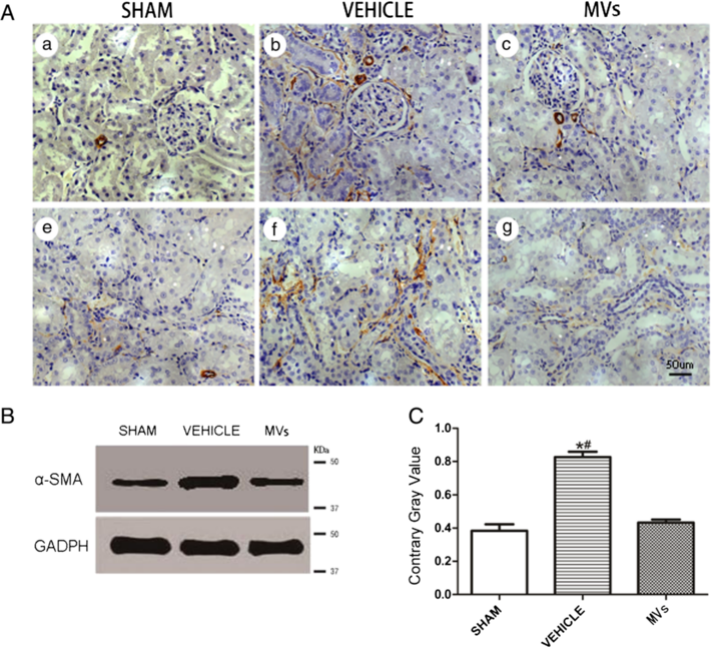
**hWJMSC-MVs reduce the α-smooth muscle actin (α-SMA) expression in kidney tissues. (A)** Representative micrographs illustrating α-smooth muscle actin (α-SMA) expression in kidney tissues, In comparison with MVs group, the vehicle group exhibited stronger positive staining for α-SMA in kidney tissue sections at two weeks (the original magnification is 400×, Scale bar = 50 um). **(B, C)** α-SMA expression in kidney tissues was measured by Western blot analysis, and MVs dramatically reduce the α-SMA expression, all quantitative data are shown as mean ± SD of three experimental conditions (n = 3, *P <0.05, sham versus vehicle; *#P* <0.05, MVs versus vehicle).

At two weeks post-IRI, the exposure to IRI led to the development of significant fibrotic lesions in the renal interstitial area, as indicated by Masson’s tri-chrome staining. In contrast, after MVs therapy there was only a slight fibrogenesis (Figure 3A,B). In addition, α-SMA expression on tubular cells, an indicator of epithelial-mesenchymal transition, occurred mostly in vehicle-treated animals and slightly in MVs infusion rats (Figure 4). Furthermore, after the right uninjured kidney was removed on Day 12, the level of serum creatinine in animals receiving vehicle infusion was significantly higher than that of sham and MV infusion on Day 14 (vehicle versus MVs, 8.30 ± 0.37 versus 4.32 ± 2.10 mg/mL, *P* <0.05; vehicle versus sham, 8.30 ± 0.37 versus 4.12 ± 0.59 mg/mL, *P* <0.05), there is no difference in 24 h and 48 h without the removal of the right uninjured kidney (Figure 3C). A similar result was observed when BUN in plasma was assessed (vehicle versus MVs, 43.81 ± 5.20 versus 25.72 ± 3.52 mg/mL, *P* <0.05; vehicle versus sham, 43.81 ± 5.20 versus 21.91 ± 5.78 mg/mL, *P* <0.05) (Figure 3D).

### The hWJMSC-MVs infusion reduced the infiltration of macrophages and attenuated inflammation in early stage

**Figure 5:**
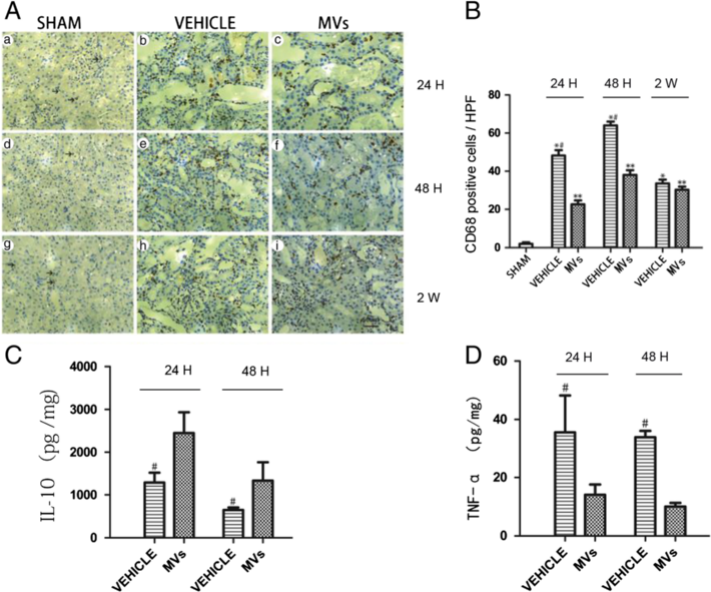
**Effects of hWJMSC-MVs on macrophage recruitment and the cytokine expression in injured kidneys. (A)** Representative images of interstitial macrophages labeled with CD68 in the kidney on 24 h, 48 h and 2 weeks after reperfusion (the original magnification is 400×, Scale bar = 50 um). **(B)** Quantification of CD68+ macrophages/HPF in the kidney sections at different time points after reperfusion, all quantitative data are shown as mean ± SD of six kidney samples for each experimental condition (n = 6, *P <0.05, sham versus vehicle; *#P* <0.05, MVs versus vehicle; **P <0.05, sham versus MVs) **(C)** ELISA analysis of the anti-inflammatory cytokine IL-10 in injured kidney at 24 h and 48 h after reperfusion, (n = 3, *#P* <0.05, MVs versus vehicle). **(D)** ELISA analysis of the TNF-α in the injured kidney at 24 h and 48 h after reperfusion. All data are shown as mean ± SD of three kidney samples for each experimental condition. (n = 3, *#P* <0.05, MVs versus vehicle). HPF, high power fields; hWJMSC-MVs, human Wharton-Jelly MSCs derived microvesicles.

The immunohistochemistry (IHC) of CD68 showed the expression of macrophages in injured kidney tissues. The IRI rat kidney had a greater number of CD68-positive macrophages than the Sham at 24 h, 48 h and 2 weeks (respectively, 52.6 ± 3.1 versus 2.0 ± 0.8 *P* <0.05; 64.4 ± 2.0 versus 2.0 ± 0.8 *P* <0.05; 36.2 ± 1.7 versus 2.0 ± 0.8 *P* <0.05; 26.1 ± 4.4 versus 2.0 ± 0.8 *P* <0.05; 38.2 ± 2.8 versus 2.0 ± 0.8 *P* <0.05; 33.6 ± 1.9 versus 2.0 ± 0.8 *P* <0.05, per high-powered field 400×), while compared to the vehicle, MVs treatment significantly decreased the infiltration at 24 h and 48 h (respectively, 26.1 ± 4.4 versus 52.6 ± 3.1 *P* <0.05; 38.2 ± 2.8 versus 64.4 ± 2.0 *P* <0.05; per high-powered field 400×). However, two weeks after reperfusion, there was no significant difference between the vehicle group and MV group (vehicle versus MVs, 36.2 ± 1.7 versus 33.6 ± 1.9, per high-powered field 400×) (Figure 5A,B). Meanwhile, the ELISA analysis of IL-10, an anti-inflammation factor, in injured kidney tissues showed an obviously elevated level after MV treatment at 24 h and 48 h (sham versus vehicle 213 ± 106 versus 1,317 ± 193 pg/mg *P* <0.05; 213 ± 106 versus 617 ± 63 pg/mg, *P* <0.05; MVs versus vehicle 2,460 ± 478 versus 1,317 ± 193 pg/mg, *P* <0.05; 1,398 ± 435 versus 617 ± 63 pg/mg, *P* <0.05) (Figure 5C). In contrast, the pro-inflammation factor TNF-α level in kidney tissues was lowered by MVs at 24 h and 48 h (sham versus vehicle, 4.7 ± 1.6 versus 38.3 ± 17.3 pg/mg, *P* <0.05; 4.7 ± 1.6 versus 36.6 ± 3.1 pg/mg, *P* <0.05; MVs versus vehicle, 16.4 ± 5.2 versus 38.3 ± 17.3 pg/mg, *P* <0.05; 14.7 ± 2.1 versus 36.6 ± 3.1 pg/mg, *P* <0.05) (Figure 5D).

### The down-regulated expression of CX3CL1 by MVs both *in vivo* and *in vitro*

**Figure 6:**
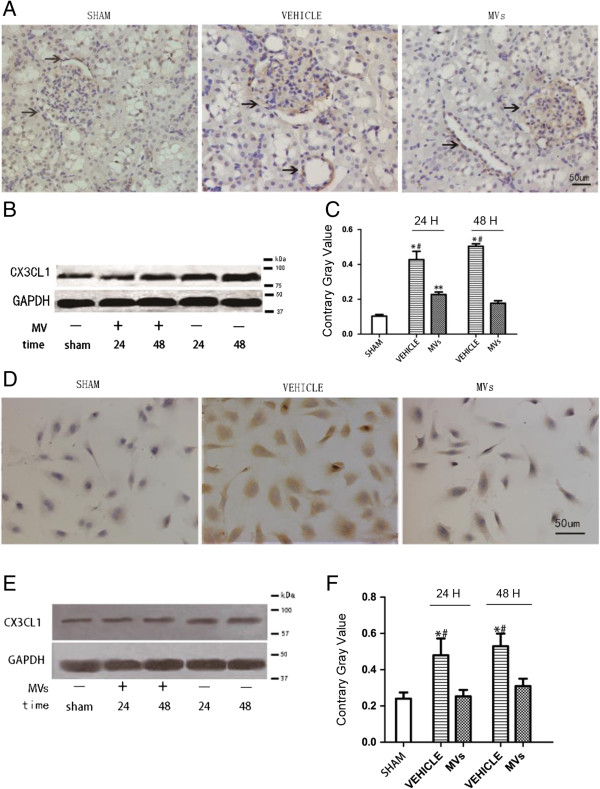
**Effects of hWJMSC-MVs administration on expression of CX3CL1 in injured kidney *in vivo *and HUVECs *in vitro*. (A)** IHC localization of CX3CL1 in the kidney 48 h after IRI. CX3CL1 was mainly expressed on endothelial cells (arrow points to positively stained endothelial cells; the original magnification is 200×, Scale bar = 50 um). **(B, C)** CX3CL1 expression in kidney tissues was measured by Western blot analysis, and MVs dramatically decreased the CX3CL1 expression. Data are expressed as mean ± SD of three experiments (n = 3, *P <0.05, sham versus vehicle; *#P* <0.05, MVs versus vehicle; **P <0.05, sham versus MVs). **(D)** ICC of CX3CL1 in HUVECs 48H after hypoxia injury (the original magnification is 200×, Scale bar = 50 um). **(E, F)** CX3CL1 expression in HUVECs was measured by Western blot analysis, and MVs dramatically decreased the CX3CL1 expression both in 24 h and 48 h after hypoxia. Data are expressed as mean ± SD of the three experiments (n = 3, *P <0.05, sham versus vehicle; *#P* <0.05, MVs versus vehicle). hWJMSC-MVs, human Wharton-Jelly mesenchymal stem cell derived microvesicles; IHC, immunohistochemistry; MVs, microvesicles.

The suppression effect of MVs on CX3CL1 was shown by IHC as well as the Western blot analysis method. As shown in Figure 6A-C, the CX3CL1 protein in the injured kidney was up-regulated compared with the sham group, but down-regulated at 24 h and 48 h after MV intervention. There was a similar observation in IHC results, which locate CX3CL1 protein at endothelial cells in glomerulus and vessels. In order to confirm the effects *in vitro*, we also performed Western blot and immunocytochemistry on HUVEC under the hypoxia-injured model. The results indicated the same as *in vivo* (Figure 6D-F). HUVEC, Human umbilical vein endothelial cells; IHC, immunohistochemistry; MVs, microvesicles.

### Profiling of CX3CL1 related micro-RNAs in hWJMSC-MVs by real-time quantitative PCR

**Figure 7:**
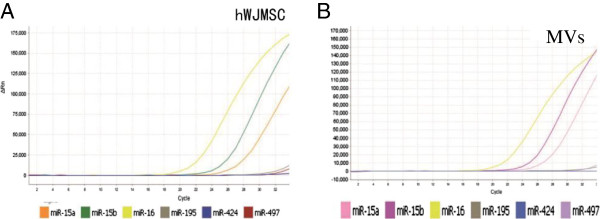
**Representative quantitative reverse transcriptase qRT-PCR for CX3CL1 related micro-RNAs in hWJMSCs and hWJMSC-MVs. (A, B)** qRT-PCR analysis of CX3CL1 related micro-RNAs in hWJMSCs and hWJMSC-MVs, data show that both the hWJMSCs and hWJMSC-MVs contain relatively high levels of miR-16, miR-15b and miR-15a, and low levels or no miR-195, miR-424 and miR-497. Each experiment was repeated three times. hWJMSC-MVs, human Wharton-Jelly mesenchymal stem cell derived microvesicles; MVs, microvesicles.

In order to investigate whether some micro-RNA contained in the MVs played a role, we searched CX3CL1 in the TargetScan database (version 6.2) and identified six putative micro-RNA (miR-15a, miR-15b, miR-16, miR-195, miR-424 and miR-497) targets to CX3CL1 mRNA by matching the seed regions of each. The real-time quantitative PCR analysis indicated that there were relatively high levels of miR-15a, miR-15b and miR-16 contained in hWJMSC-MVs, but none or low levels of miR-195, miR-424 and miR-497 (Figure 7B). The same results were seen in hWJMSCs (Figure 7A).

## Discussion

In the present study, we observed that a single administration of hWJMSC-MVs immediately after induction of IRI could decrease the expression of CX3CL1 in the kidney and lessen the infiltration of CD68+ macrophages at the 24 h and 48 h time points after the reperfusion. Meanwhile, the blood vWF level, a parameter reflecting the injury to endothelial cells, declined and the apoptosis of renal cells mitigated in the early stage of renal IRI. Furthermore, the renal function was ameliorated and kidney fibrosis was abrogated by MVs in the late stage.

In recent years, strategies for administration of stem cells from various sources, such as bone marrow, fetal membrane and adipose, for repairing kidney injury have been reported [26-28], but the potential mechanisms remain unclear. In previous studies, Caplan *et al*. have suggested that MSCs may provide a paracrine support for kidney repair [17]. Consistently, Bi *et al*. demonstrated that the administration of conditioned medium from MSCs may also have beneficial effects on AKI [20], indicating that endocrine or paracrine mechanisms play an important role. Our group also found that hWJMSCs could alleviate acute and chronic kidney injury through an endocrine mechanism [29]. MVs, which are contained in the condition medium of MSCs, are small vesicles released by cells bearing the surface antigens characteristic of the original cells. They can also enter the target cells through specific receptor ligand interactions and transfer micro-RNA, mRNA, proteins and other bioactive materials [21,30-32]. Bruno *et al*. demonstrated a protective effect of bone marrow MSC-derived MVs on acute kidney injury and found that MVs could be detected in an injured kidney after intravenous administration [21]. In the present study, we isolated MVs from hWJMSC-conditioned medium, characterized them successfully and found that hWJMSC-MVs have a crucial therapeutic effect in AKI.

To investigate the potential mechanisms of MVs in the IRI, we detected the CD68+ macrophages in the injured kidney. We found that the expression of CD68+ macrophages as well as the serum level of vWF in the MVs group declined at 24 h and 48 h after reperfusion. Macrophages’ effects on tissue repair depend on their phenotypes and the inflammatory milieu [33,34]. Some strategies to block the initial inflammatory response, such as depleting macrophages or inhibiting the action of inflammatory cytokines, are protective against ischemic AKI [9,33]. In our study we found CD68+ macrophages were reduced significantly by MVs in the early stage of AKI. Inflammatory cytokines are known to play an important role in ischemic AKI; the decreased expression of TNF-α and increased expression of IL-10 in the injured kidney was also observed. Moreover, the renal cell apoptosis was mitigated and proliferation was enhanced in the MV group. From the data above, we confirmed that hWJMSC-MVs play a therapeutic role in I/R-induced acute kidney injury through the regulation of inflammation.

To further study the potential mechanisms, we evaluated the expression of CX3CL1, which is a potent chemo-attractant protein for macrophages. We found that the expression of CX3CL1 was up-regulated obviously in the kidney after ischemic AKI, which is in accordance with the published data [13,16]. Interestingly, in the MV group, the CX3CL1 was dramatically down-regulated in the injured kidney. Further, in *in vitro* studies, we also found that MVs could suppress the expression of CX3CL1 in cultured HUVECs under a hypoxia-oxygen injury model. On the other hand, previous studies show that CX3CL1 with its cognate receptor CX3CR1 could mediate the immune cell adhesion in the pathogenesis of vascular injury [35,36]; the authors also found that the expression of CX3CL1 protein in the kidney increased markedly in ischemic AKI and that CX3CL1/CX3CR1 promotes kidney injury both in hypertensive and ischemic animal models [15,16]. Meanwhile, inhibition of CX3CR1, the only known receptor of CX3CL1, could reduce the macrophages in an injured kidney and have therapeutic effects in AKI [14,16]. Thus, we concluded that down-regulation of CX3CL1 by MVs might be a way to reduce the infiltration of macrophages in the kidney and MVs’ inflammation regulation property may be one of the underlying mechanisms of hWJMSC-MVs’ therapeutic effects.

It has also been shown that RNA, including mRNA and micro-RNA, contained in the MVs could be transferred into the target cells and may play an important role in the repair of I/R-induced AKI [31]. Recently, some studies also found that different cell-derived MVs protect against acute kidney injury via a horizontal transfer of related mRNAs or micro-RNAs [21,37]. Thus, we supposed that some secreted patterns of micro-RNAs, which can modulate the expression of CX3CL1, may be included in the hWJMSCs-MVs. Moreover, it has also been reported that some micro-RNAs predicted by the Targetscan Database for particular molecule was confirmed [38]. So we paid more attention to miR-15a, miR-15b, miR-16, miR-195, miR-424 and miR-497, which are the targeted results for CX3CL1 in the Targetscan Database. This means these micro-RNAs are the biological target molecules and, by matching the CX3CL1 to the seed region, may have the potential effects of modulation of CX3CL1. Interestingly, the miR-16, miR-15b and miR-15a were profiled successfully both in hWJMSCs and hWJMSC-MVs by the real-time quantitative PCR method with a high concentration, and we think that these micro-RNAs contained in MVs may involve the modulation of CX3CL1 expression. However, how MVs modulate the expression of CX3CL1 and whether these micro-RNAs have functional effects are unknown. We plan to investigate the deeper mechanism in our next project.

## Conclusions

A single administration of hWJMSC-MVs immediately after IRI could ameliorate kidney ischemia/reperfusion injury both in the acute and chronic stages, and it could also suppress the expression of CX3CL1 and mitigate the inflammation levels in injured kidneys in the early stage, which may be regarded as a novel mechanism for therapeutic effects on ischemic AKI.

## Abbreviations

α-SMA: alpha smooth muscle actin; AKI: acute kidney injury; BSA: bovine serum album; CM: Conditioned medium; HPF: high power fields HUVECs, human umbilical vein endothelial cells; hWJMSC-MVs: human Wharton’s Jelly mesenchymal stromal cells derived microvesicles; IL-10: interleukin 10; IRI: ischemia/reperfusion injury; MVs: microvesicles; PBS: phosphate-buffered saline; TNF-α: tumore necrosis factor alpha; TUNEL: terminal transferase-mediated dUTP nick-end labeling; vWF: von Willebrand Factor.

## Competing interests

The authors declare that they have no competing interests.

## Authors’ contributions

XYZ and GYZ participated in performing the animal experiments and collecting data, and in conception and design, data analysis and interpretation, manuscript writing and revision. ZLC, DMY and TD performed animal experiments, helped with conception and design, and did manuscript revision. GQJ, SM and GHL performed cell culture, data analysis and interpretation, and manuscript revision. YJZ and MJL were involved in obtaining financial support, in conception and design, and manuscript revision. All authors read and approved the final manuscript.

## Supplementary Material

Additional file 1: Figure S1 Distribution of hWJMSC-MVs after injection. Representative confocal micrographs of frozen tissue sections of rats injected with PKH26-labeled MVs (red) kidneys. Red fluorescence (white arrows) was observed in the kidneys after injection of PKH26-labeled MVs at 3 h. Tubular epithelial cell cytoplasm and nuclei were stained green and blue, respectively. (Original magnification 200 ×). Click here for file
