# Supplementary material for: Microvesicles derived from human Wharton’s Jelly mesenchymal stromal cells ameliorate renal ischemia-reperfusion injury in rats by suppressing CX3CL1
Source: Stem Cell Res Ther. 2014 Mar 19;5(2):40. doi: 10.1186/scrt428 (PMC4055103; doi:10.1186/scrt428)
Supplement: Additional file 1: Figure S1 — Distribution of hWJMSC-MVs after injection. Representative confocal micrographs of frozen tissue sections of rats injected with PKH26-labeled MVs (red) kidneys. Red fluorescence (white arrows) was observed in the kidneys after injection of PKH26-labeled MVs at 3 h. Tubular epithelial cell cytoplasm and nuclei were stained green and blue, respectively. (Original magnification 200 ×). [file scrt428-S1.pdf]

Additional file 1: Figure S1

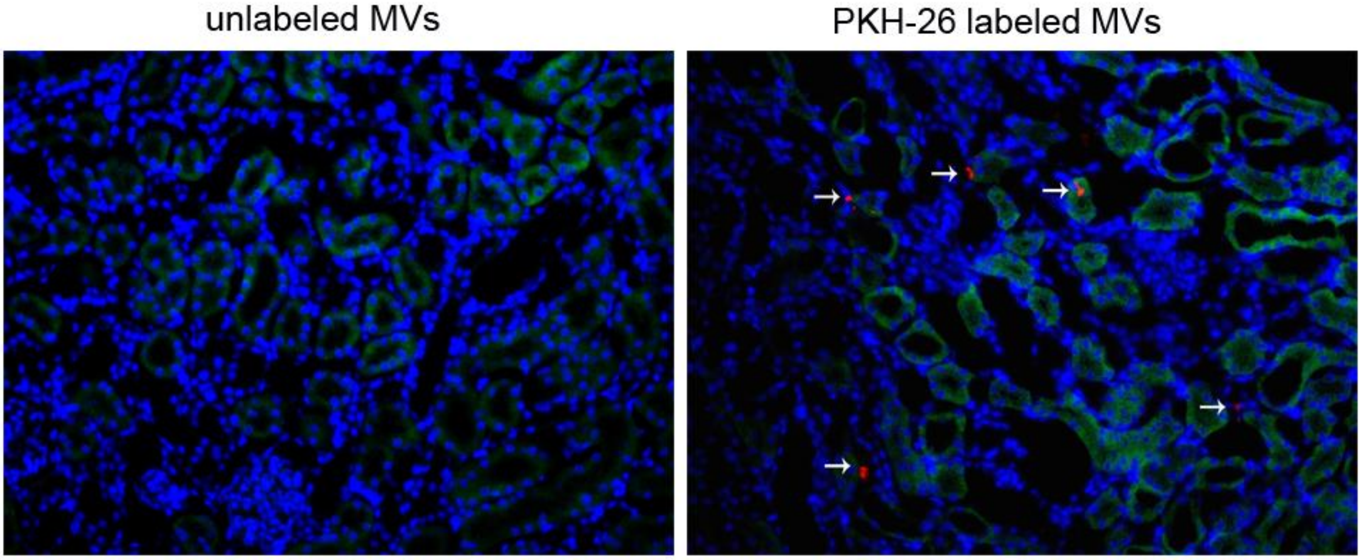

Figure.S1 Distribution of hWJMSC-MVs after injection.

Representative confocal micrographs of frozen tissue sections of rats injected with PKH26-labeled MVs (red) kidneys. Red fluorescence (white arrows) was observed in the kidneys after injection of PKH26-labeled MVs at 3 h. Tubular epithelial cell cytoplasm and nuclei were stained green and blue respectively. Original magnification ×200.
